# Improving the Oral Health of Older People in Care Homes: Results From a Randomised Feasibility Study

**DOI:** 10.1111/cdoe.13043

**Published:** 2025-06-10

**Authors:** Georgios Tsakos, Paul R. Brocklehurst, Saif Syed, Michelle Harvey, Sana Daniyal, Sinead Watson, Nia Goulden, Anna Verey, Peter Cairns, Anja Heilmann, Zoe Hoare, Frank Kee, Joe Langley, Nat Lievesley, Ciaran O'Neill, Andrea Sherriff, Craig J. Smith, Rebecca R. Wassall, Richard G. Watt, Gerald McKenna

**Affiliations:** ^1^ Institute of Epidemiology and Health Care University College London London UK; ^2^ Public Health Wales Cardiff UK; ^3^ Centre for Public Health Queens University Belfast Belfast UK; ^4^ North Wales Organisation for Randomised Trials in Health (NWORTH) Bangor University Bangor UK; ^5^ Institute of Psychiatry, Psychology, and Neuroscience King's College London London UK; ^6^ Public Contributor (PPI) UK; ^7^ North Wales Medical School Bangor University Bangor UK; ^8^ Lab4Living Sheffield Hallam University Sheffield UK; ^9^ School of Medicine University of Glasgow Glasgow UK; ^10^ Division of Cardiovascular Sciences University of Manchester Manchester UK; ^11^ School of Dental Sciences Newcastle University Newcastle UK

**Keywords:** care homes, cluster randomised controlled trial, complex intervention, feasibility study, older adults, oral health

## Abstract

**Objectives:**

Poor oral health is a considerable burden for older adults in care homes. The National Institute for Health and Care Excellence (NICE) issued guideline NG48 on “Improving oral health in care homes”. However, empirical evidence for oral health interventions among care home residents is weak, and the feasibility of the NG48 recommended interventions is not established. This study aimed to determine the feasibility of delivering a co‐designed oral health intervention, based on NG48 recommendations, in care homes in two sites in the UK.

**Methods:**

This was a pragmatic cluster randomised controlled feasibility study with a 12‐month follow‐up, undertaken in 22 care homes across two sites (11 each in London and Northern Ireland). Care homes were randomised to an intervention arm (*n* = 11), and a control arm (*n* = 11) that continued with usual routine practice. The complex intervention contained materials were co‐designed with care home staff and consisted of: care home staff training package; Oral Health Assessment Tool (OHAT) administered by trained care home staff; and a support worker assisted twice daily tooth‐brushing regimen with 1500 ppm fluoride toothpaste. Rates of recruitment and retention, data completion, and intervention fidelity were recorded to determine feasibility.

**Results:**

One‐hundred‐and‐nineteen residents from 22 care homes were recruited and 82 residents from 19 care homes completed the study (retention: 86% for care homes and 69% for residents). Twenty residents were lost to follow‐up and another 17 withdrew throughout the study. Data completion rates ranged between 88% and 97% at baseline and between 91% and 96% at the 12‐month follow‐up. Intervention fidelity records showed high completion rates for oral care plans (90%), and lower rates for weekly oral hygiene records (73%) and the OHAT (61%).

**Conclusions:**

This study documented the feasibility of an oral health intervention in care homes, while also highlighting issues to consider for a definitive trial to assess the effectiveness of the co‐designed intervention.

**Trial Registration:**

Clinical Trial Registration: ISRCTN10276613

## 1 Introduction

Changes in the epidemiological profile of older adults in the United Kingdom (UK) mean that the vast majority are dentate, with heavily restored dentitions, where poor oral health and excessive treatment needs are increasingly common [1]. Approximately 400 000 older people live in care homes across the UK, representing 4% of the total population aged 65 years and older, and 15% of those aged 85 years and older [2]. The oral health of care home residents is considerably worse than their peers living in the community, with a much higher prevalence of caries and periodontal diseases among the dentate [3], who constitute the majority of residents [4]. The high burden of oral conditions among care home residents is a common feature across countries in Europe and for different health care systems [5].

Poor oral health impacts negatively on older adults' general health, diet and nutrition [6, 7, 8] and quality of life [9, 10]. Whilst good daily oral hygiene is essential to prevent chronic dental diseases, with increasing age, the ability to sustain good oral health can deteriorate. For older adults this is often exacerbated by the burden of cumulative multimorbidity, cognitive impairment and frailty [11, 12, 13, 14, 15, 16], while polypharmacy leads to negative impacts associated with xerostomia [17, 18]. For care home residents who cannot access primary dental care in the UK, access to domiciliary services is often difficult [19, 20]. Residents' diets can become rich in sugars [21, 22], especially for those with a diminished appetite who rely on sugar to provide additional calories to manage or prevent malnutrition and frailty. All these factors increase the risk of oral diseases and comorbidities, impacting on oral and general health.

In 2016, the National Institute for Health and Care Excellence (NICE) issued guideline NG48 [23] to improve the oral health of care home residents. The guideline recommended that policies on oral health should be developed and followed, although the evidence base was also recognised as weak. It is advised that residents have their mouths assessed by care home staff, who should have the knowledge and skills to support people's oral health, and care plans should include daily mouth care, while residents should also have access to local dental services. However, a Care Quality Commission review in England in 2019 illustrated an extensive lack of awareness of the NG48 and showed that most care homes had no oral health policy, with nearly half of staff not receiving any training in daily oral healthcare, and very few care homes had access to routine or emergency dental care [19].

The provision of daily oral care practices in care homes is complex and challenging, commonly heterogeneous and includes intermittent tooth brushing by the residents, who usually rely on support from care home staff for their oral hygiene [24, 25, 26, 27, 28]. Empirical evidence on the effectiveness of oral health interventions among care home residents is limited and weak [24, 29]. A recent systematic review [30] highlighted concerns around study quality, with only a handful of experimental studies having a low risk of bias, while the evidence from better quality studies was inconclusive, with some studies reporting that interventions led to improved oral health [31, 32, 33] while others did not [34, 35]. Only one randomised clinical trial tested the effectiveness of an oral health guideline [36] and this was judged to be unclear in terms of risk of bias. No research has assessed whether the NG48 recommended interventions are feasible in a UK setting. There is also uncertainty about the recruitment and retention of residents, intervention fidelity, and appropriate outcome measures. Moreover, the NG48 in its original format was found to be of little practical value as it did not provide guidance to address the practical challenges that care home staff face for the provision of oral health care to residents [28]. To address this, practical tools to support the implementation of key NG48 aspects were co‐designed together with care home staff [37]. The aim of this study was to determine the feasibility of delivering a co‐designed oral health intervention, based on NG48 recommendations, in care homes in two sites in the UK. Being a feasibility study, the focus was on recruitment, retention, and fidelity: the proportion of care homes and residents who were eligible and willing to participate; the proportion of care homes and residents that received the intervention and completed the study; and the proportion of completed data. A theoretically informed process evaluation [38] and a feasibility of the respective cost and consequences [39] were conducted in parallel.

## 2 Methods

Ethical approval was received from the London: City & East Research Ethics Committee (ref: 19/LO/1107). All participants provided written informed consent.

Recruitment was a two‐stage process. Care homes were informed about the study, in collaboration with the Noclor NHS Research Office, Clinical Research Networks (North Thames and North West London) and the ENRICH network, and with the Whittington Health NHS Trust Dental Services in London and the South Eastern Health and Social Care Trust in Northern Ireland. If interested, the research team provided the care home managers with an information sheet and further discussed the study, and answered questions. Care homes were eligible to participate if they had capacity for at least 20 residents. Care homes were excluded if they only had high‐dependency units or provided end‐of‐life care.

The second stage involved screening and recruitment of eligible residents in participating care homes. Residents were eligible to participate if they had capacity to provide consent and met the following inclusion criteria: aged 65 years and over; dentate or partially dentate (as a key element of the intervention relates to assisted tooth brushing); and living full‐time in the care home. Residents were not eligible if they: were receiving end‐of‐life or palliative care; had severe cognitive impairment (6‐item Cognitive Impairment Test (6‐CIT) score of 10 or higher) [40, 41]; or did not have a working level of spoken English.

The screening process for residents comprised three steps. In step one, the care home manager/staff identified potentially eligible residents, provided them with a Participant Information Sheet (PIS) and asked them to consent for two eligibility tests. Once informed consent for eligibility testing was obtained, a researcher administered the 6‐CIT test (step two). Residents with no or mild cognitive impairment (6‐CIT score ≤ 9) were potentially eligible for the study. In step three, a researcher confirmed whether the resident was dentate or partially dentate by performing a brief dental check. If the resident was eligible, the researcher provided a PIS and later asked the resident to complete the informed consent form for participation in the feasibility study.

A sample of 120 participants would allow an estimation of the projected attrition rate of 20% to within a 95% confidence interval of ± 7% [42]. To achieve this sample, 22 care homes (11 each in London and Northern Ireland) were recruited between December 2021 and October 2022. Care homes were randomly allocated to the intervention and control arms on a 1:1 ratio and stratified by location (London/Northern Ireland), using a dynamic adaptive randomisation algorithm [43]. Due to the nature of the complex intervention, blinding of care homes and residents was not feasible. The clinical dental examiners and the trial statistician were blinded to the allocation of care homes into study arms.

Care homes allocated to the intervention arm implemented for 12 months a complex intervention based on NG48 recommendations [23]. The intervention [44] consisted of:

1. A care home staff training package (training video, hard copy training manuals, laminated reference guides, as well as online training) to facilitate appropriate knowledge and skills to implement oral health promotion activities. Staff were required to undertake the training, overseen by the care home manager and added to the mandatory training log, prior to the intervention. They had access to the training package, in both a hard copy and online format, throughout the duration of the study and it was also used in the induction training for new staff.
2. Administration by trained care home staff of the Oral Health Assessment Tool (OHAT) [45] at baseline and the 12‐month follow‐up visit. Following completion of the OHAT at baseline, staff were asked to complete a ‘Personal Oral Care Plan’ for each resident and update it after reassessment or any dental visit.
3. A support worker assisted twice‐daily (morning and evening) tooth‐brushing regimen using 1500 ppm fluoride toothpaste. This involved staff assisting in or brushing the teeth of residents who had difficulty with their oral self‐care.

The complex intervention was adapted through a co‐design process working with care home staff, with a suite of materials produced (‘Oral Health Assessment Tool’, ‘Personal Oral Care Plan’, ‘Tips and tricks’ for care home staff, and ‘Weekly Oral Hygiene Record’) [37].

Care homes allocated to the control arm were asked to continue with their usual routine practice during the same 12‐month period.

Pertinent to a feasibility study, the outcomes [44] were to determine the:

1. Proportion of care homes that agreed to participate.
2. Number of residents that were eligible and able to consent.
3. Proportion of eligible residents that agreed to participate.
4. Proportion of participating residents that received the intervention per the protocol.
5. Proportion of care homes and residents that remained in the study (75% target).
6. Proportion of completed data for measures used (75% target).
7. Impact on recruitment of varying the cognitive impairment eligibility threshold.

Different oral health variables were also measured:

1. Clinical oral health (assessed at baseline and 12‐months by two trained dental examiners), referring to the number of teeth present, the number of teeth with coronal caries, the number of teeth with root caries, the proportion of teeth with visible plaque, and the proportion of teeth that bled on probing.
2. Oral health needs, assessed through the OHAT [45] (collected by the dental examiners at baseline and 12‐months).
3. Oral health‐related quality of life, using the Oral Impacts on Daily Performances (OIDP) [46] questionnaire (administered by trained researchers to residents at baseline, 6‐ and 12‐months). The OIDP is validated among older adults in the UK [46], previously used in care homes [9], and included in the national adult oral health surveys [47].
4. Oral symptoms and urgent dental care referrals (collected weekly by care home staff and at baseline, 6‐ and 12‐months by the researchers), consisting of the number of reported episodes of dental pain, sepsis, discomfort, and urgent dental care appointments.

### 2.1 Data Analysis

The statistical analysis plan was approved by the Data Monitoring and Ethics Committee and the Study Steering Committee. Commensurate with a feasibility study, analysis was restricted to generating summary statistics and confidence intervals. Primary analysis focused on feasibility outcomes. Recruitment and retention outcomes with associated estimates of precision were summarised. Preliminary exploratory analysis of the oral health measures was undertaken considering potential to measure change, understanding measure behaviour, and appropriateness to the population. All statistical analyses were undertaken at NWORTH CTU on an intention to treat basis accommodating the clustering of participants within care homes. No interim analyses were planned, and no missing data were imputed.

For oral health variables, appropriate exploratory multilevel mixed effect models were fitted examining the 12‐month measure as the dependent variable, incorporating the baseline variable as a covariate, allocated study arm and site as factors and care home as a random effect. A linear model was employed for the OHAT, including Kenward‐Roger correction for small degrees of freedom, and a negative binomial regression model for the number of teeth with coronal and root caries and the OIDP. The proportion of teeth that bled on probing and the proportion of teeth with visible plaque were analysed using a fractional response regression as above but including the care home as a factor rather than a random effect. Cluster‐robust standard errors were utilised for this analysis.

## 3 Results

**FIGURE 1:**
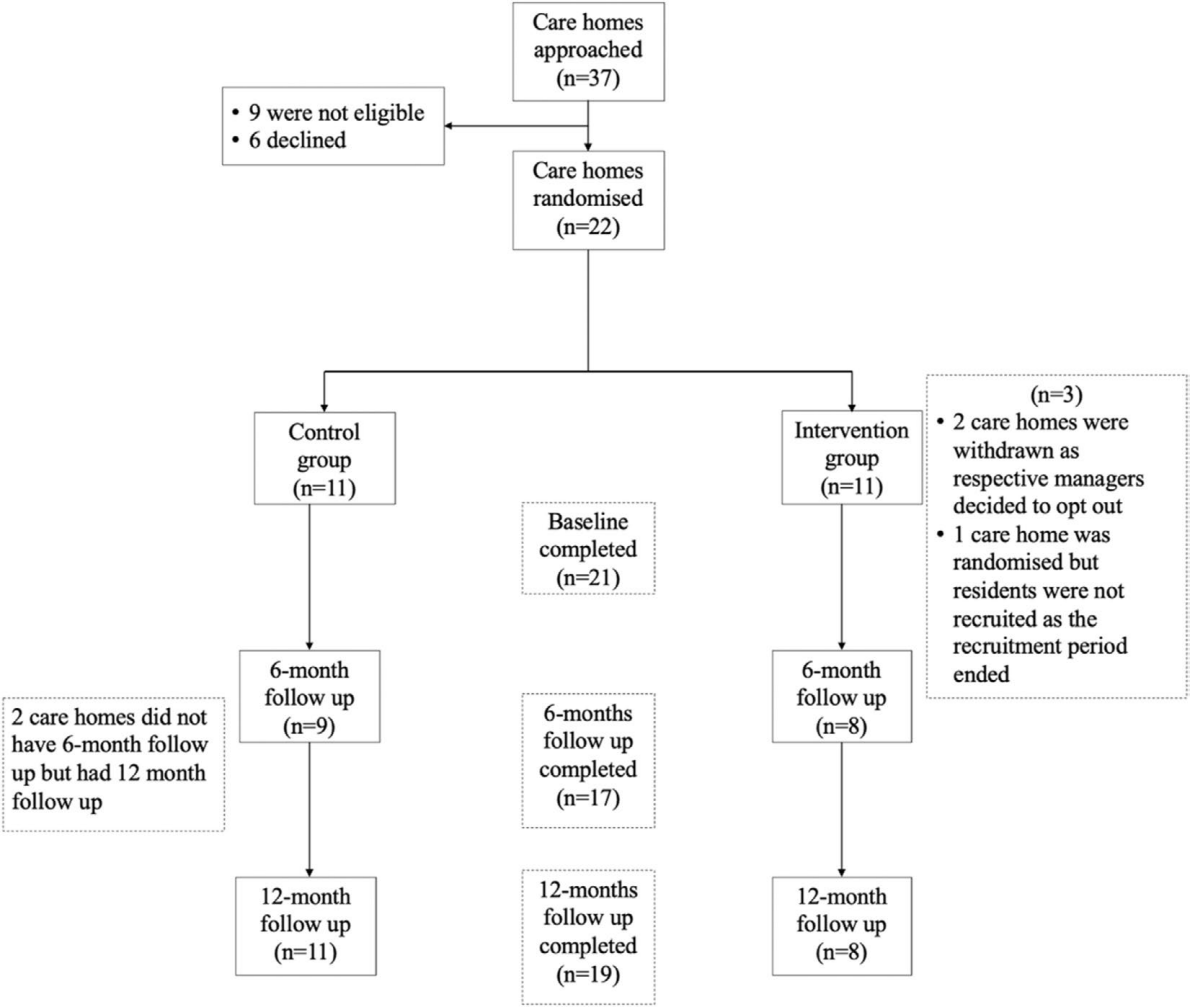
Care homes recruitment and retention in the TOPIC study.

**FIGURE 2:**
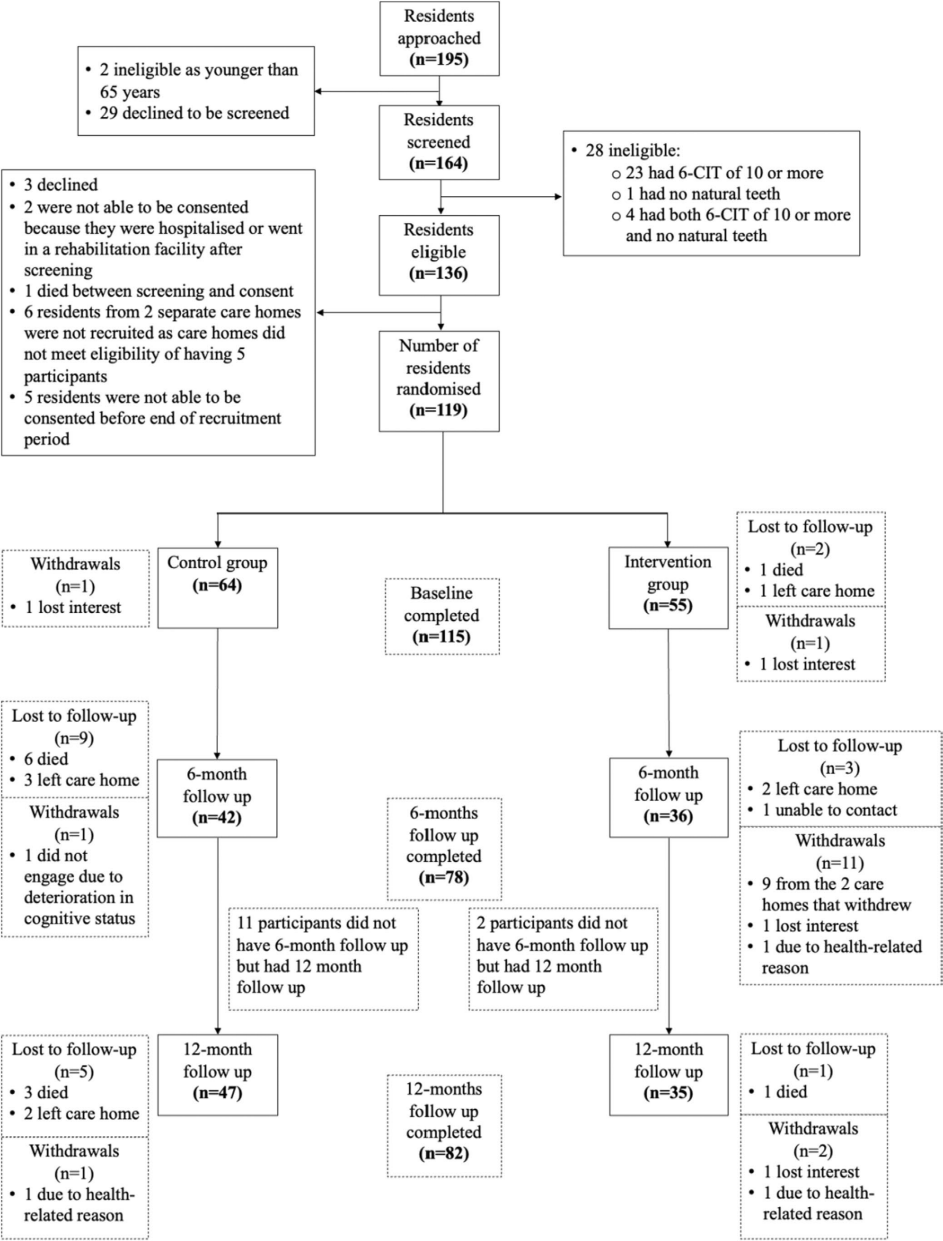
Residents' recruitment and retention in the TOPIC study.

Thirty‐seven care homes were invited to participate, and 31 (84%) agreed. Of those, 9 were ineligible, and the remaining 22 were recruited and randomised; 11 care homes each to the control and intervention arms. Seventeen care homes completed the 6‐month follow‐up, and nineteen care homes (86% of those recruited) remained in the study and completed the 12‐month follow‐up (Figure 1). In terms of recruitment and retention of residents, 195 were initially approached, 164 underwent eligibility screening, and 136 residents were eligible and able to consent. Seventeen residents were not recruited for different reasons, meaning 119 residents were recruited to the study (88% recruitment rate); randomised 64 to the control arm and 55 to the intervention arm (Figure 2). Of the 119, two residents were lost from the study prior to baseline assessments, and another two withdrew; therefore, baseline assessments were completed for 115 residents. At the 6‐month follow‐up, another 12 residents were lost to follow‐up, and a further 12 residents had withdrawn. At the 12‐month follow‐up, 6 more residents were lost to follow‐up, and a further 3 had withdrawn. Thirteen residents did not complete the 6‐month follow‐up but did complete the 12‐month follow‐up data. Overall, 82 residents (from the 119 recruited), 47 in the control and 35 in the intervention arm, completed the 12‐month follow‐up (69% retention rate) (Figure 2).

Overall, 105 residents (88% of the 119 recruited) had fully completed the clinical oral health and the researcher‐administered questionnaire data at baseline, and 75 residents at the 12‐month follow‐up (91% of the 82 retained in the study, and 63% of the 119 initially recruited). Completion rates for the different categories of data collected by the research team ranged between 88% and 97% at baseline and between 91% and 96% at the 12‐month follow‐up (Table 1). There were more missing data for the weekly oral symptom checklists that were completed by care home staff; 92 residents (77% of 119 recruited) had at least one weekly checklist completed, but only two residents had complete checklist data for all weeks. In terms of intervention fidelity records, 90% (45 out of 50) of the intervention arm residents had fully or partially completed oral care plans, while 73% (1243 out of 1708) of weekly oral hygiene records and 61% (62 out of 102) of the OHAT records administered by the care home staff were completed.

**TABLE 1:** Residents' data completion numbers and rates: baseline and 12‐months follow‐up.

Data category^a^	Baseline (*n* = 119)		12‐months follow‐up (*n* = 82)	
	Number	Rate (%)	Number	Rate (%)
Clinical examination	107	90%	79	96%
Oral health assessment tool	105	88%	75	91%
Oral health related quality of life (OIDP)	115	97%	77	94%
Oral symptoms checklist	115	97%	78	95%

^a^ Data were collected by the research team.

Descriptive characteristics of the sample at baseline are in Supplementary Table S1. The mean age was 83.6 years, 59% were female, and 86% identified themselves as English/Welsh/Scottish/Northern Irish/British/Irish. Eighty‐eight residents (77%) had no cognitive impairment (6‐CIT score of 0–7), while the remaining 27 had mild cognitive impairment (6‐CIT scores of 8–9).

The differences between intervention and control arms were generally modest for the different oral health measures, in both baseline and 12‐month follow‐up (Table 2). This pattern was also evident in multilevel multivariable analyses. Relatively larger differences were observed for bleeding on probing, with the adjusted proportion of teeth that bled on probing at the 12‐month follow‐up being 58% (95% CI: 45%, 71%) for the control and 49% (95% CI: 31%, 69%) for the intervention arm (Table 3), and for oral health‐related quality of life, with the intervention arm having lower OIDP score, indicating better quality of life, than the control arm (prevalence ratio: 0.80, 95% CI: 0.17, 3.67) (Table 4). The intracluster correlation coefficient (ICC) was: 0.40 for the OIDP; 0.32 for the proportion of teeth that bled on probing; 0.04 for the number of teeth with root caries; 0.02 for the OHAT; ICC for all other measures was less than 0.01.

**TABLE 2:** Oral health measures at baseline and 12‐months follow‐up: number of residents and mean, median or proportion (95% CI or interquartile range) for the control arm, the intervention arm and the whole sample.

		Control arm	Intervention arm	Whole sample
Coronal caries: Baseline	Number	61	46	107
	Median	0	0	0
	[IQR]	[0, 0]	[0, 0]	[0, 0]
Coronal caries: 12‐months follow‐up	Number	47	32	79
	Median	0	0	0
	[IQR]	[0, 0]	[0, 0]	[0, 0]
Root caries: Baseline	Number	61	46	107
	Median	0	0	0
	[IQR]	[0, 2]	[0, 4]	[0, 2]
Root caries: 12‐months follow‐up	Number	47	32	79
	Median	0	1	0
	[IQR]	[0, 2]	[0, 3]	[0, 2]
Bleeding on probing: Baseline	Number	61	44	105
	Proportion	0.40	0.47	0.41
	[95% CI]	[0.31, 0.49]	[0.34, 0.60]	[0.33, 0.49]
Bleeding on probing: 12‐months follow‐up	Number	45	32	77
	Proportion	0.55	0.54	0.54
	[95% CI]	[0.44 0.66]	[0.40 0.68]	[0.46 0.63]
Plaque: Baseline	Number	61	44	105
	Proportion	0.57	0.63	0.59
	[95% CI]	[0.46, 0.68]	[0.51, 0.76]	[0.52, 0.68]
Plaque: 12‐months follow‐up	Number	45	32	77
	Proportion	0.73	0.71	0.72
	[95% CI]	[0.63 0.82]	[0.60 0.82]	[0.65 0.79]
OHAT: Baseline	Number	61	44	105
	Mean	4.4	5.4	4.8
	[95% CI]	[3.7, 5.2]	[4.5, 6.3]	[4.3, 5.4]
OHAT: 12‐months follow‐up	Number	46	29	75
	Mean	5.0	5.5	5.2
	[95% CI]	[4.2, 5.7]	[4.5, 6.5]	[4.6, 5.8]
OIDP: Baseline	Number	63	52	115
	Median	0	0	0
	[IQR]	[0, 3.5]	[0, 0]	[0, 2]
OIDP: 12‐months follow‐up	Number	46	31	77
	Median	0	0	0
	[IQR]	[0, 6]	[0, 4]	[0, 5]

**TABLE 3:** Oral health measures at 12‐months follow‐up: number and adjusted estimates from linear and fractional response analysis models for the control arm, the intervention arm and the whole sample.

		Control arm	Intervention arm	Whole sample
Bleeding on probing	Number	45	32	77
	Adjusted proportion	0.58	0.49	0.54
	[95% CI]	[0.45, 0.71]	[0.31, 0.69]	[0.39, 0.70]
Plaque	Number	45	32	77
	Adjusted proportion	0.74	0.69	0.72
	[95% CI]	[0.69, 0.80]	[0.67, 0.70]	[0.69, 0.75]
OHAT	Number	46	29	75
	Adjusted mean	5.11	5.21	5.15
	[95% CI]	[4.51, 5.71]	[4.46, 5.96]	[4.68, 5.61]

**TABLE 4:** Oral health measures at 12‐months follow‐up: adjusted prevalence ratio (95% CI) from negative binomial analysis models.

Outcome	Factor	Prevalence ratio [95% CI]
Coronal caries	Study arm (control vs intervention)	1.44 [0.31, 6.61]
Root caries	Study arm (control vs intervention)	0.94 [0.44, 2.02]
OIDP	Study arm (control vs intervention)	0.80 [0.17, 3.67]

Twenty adverse events were reported during the study, 17 of them serious (12 participants died and 5 were admitted to hospital), but all were unrelated to study participation. There were three protocol deviations, all reviewed by the Data Monitoring and Ethics Committee. One recruited care home did not have 20 residents in situ but had capacity for 20 residents. The protocol was updated to refer to care home capacity for 20 residents, which was more appropriate given resident numbers fluctuation. The second deviation occurred when a 62‐year‐old resident was recruited, with the eligibility criteria being 65 or older. The third was caused by the loss of weekly symptoms checklists for one resident.

## 4 Discussion

This study of a co‐designed oral health intervention in care homes in two settings in the UK documented the feasibility of recruitment and retention of both care homes and residents, and of successful data completion and intervention recording, thereby paving the way for a subsequent definitive trial to assess the effectiveness of the intervention.

Recruitment and retention of care homes is paramount for undertaking research in this setting. This study showed that it was possible to recruit a considerable number of care homes across two different sites for research on an intervention to improve oral health in dependent older adults. Retention of care homes was also promising, with three (out of 22) homes lost over the 12‐month period. The timing of attrition is also relevant, with two homes withdrawing from the study soon after recruitment and baseline data collection, and after changes in their management, and another one not agreeing arrangements for recruitment of residents within the set timeframe. This highlights the importance of care homes' organisational characteristics, management stability, and commitment to the study as key success factors for retention in a subsequent definitive trial. Extending the recruitment period may also be helpful, particularly considering the challenging research context.

A large number of eligible residents were identified and successfully recruited, achieving the required sample size. This process also highlighted potential logistical challenges of undertaking a study in this population group and context. Recruitment of residents in the study requires strong commitment by the care home staff [38], and repeated (weekly) follow‐up engagement of the research team with care home management and staff was important to facilitate this. Of the 195 residents approached, 119 were eventually recruited, with at least 30 residents being ineligible due to severe cognitive impairment. Adjusting the threshold for the 6‐CIT screening tool to include residents with severe cognitive impairment could ameliorate this and increase the generalisability of the findings but would also bring a level of increased complexity, particularly in terms of recruiting participants (e.g., procedures should allow for proxy consent for residents with severe cognitive impairment that would not have the capacity to consent themselves), as well as for the training of staff and intervention delivery so that the materials and techniques are tailored for the aforementioned residents. Moreover, ethical approval for the feasibility study was specifically provided only for residents without or with mild cognitive impairment; therefore, a justified argument is required to extend this to residents with severe cognitive impairment in the subsequent definitive trial. From a moral, ethical perspective, the argument for inclusion is strong, considering the increasing proportion and vulnerability of care home residents that suffer from severe cognitive impairment. By contrast, limiting the sample to those with no cognitive impairment, i.e., excluding those with mild cognitive impairment, would have reduced the recruited sample by almost a quarter while they were shown to be similar in oral health measures to the residents without cognitive impairment. Broadening inclusion criteria was also highlighted, in a recent review, as a key consideration to facilitate recruitment [48].

Sample retention is a critical methodological consideration in trials involving older adults in care homes [48]. The attrition rate at 12 months was 31% of the recruited sample and this was slightly lower than the a priori set threshold (25%). This attrition is comparable with other relevant trials in care homes, with rates of 15% [34, 49], 18% [31], 20% [33], and 32% [50] over 6 months, i.e., half the duration of this study, while in a recent study in the UK it was 26.5% over 12 months [29]. A closer look at the attrition in this study indicates that this was almost equally split between those lost to the study follow‐up (either because they died or moved out of the care home) and those who withdrew from the study, either because they lost interest, or for health reasons, or because their care home withdrew (resulting in loss of study participants). Acknowledging the size of the former group (residents who were lost to the follow‐up) and planning accordingly for a subsequent trial is important learning. For half of the latter group (residents that withdrew), this was due to the withdrawal of their care home, again demonstrating the key role of engagement with and consistent “buy‐in” to the study from care homes management. This is aligned with the key process evaluation finding that identified a hierarchical structure of influence in care homes and showed that the organisation and efficiency of processes varied considerably between homes, highlighting the role of values and beliefs of those that run the care home and those that deliver the intervention [38]. In addition, active engagement and periodical follow‐ups of the research team with the care home management and staff appeared to facilitate longer‐term retention of residents in the study.

Data completion rates were high for the data collected by the research team. This applied to clinical examinations and researcher‐administered questionnaires. However, when the responsibility for data collection fell to care home staff, as was the case with the weekly symptoms checklist, data completion rates were poor. Consideration should be given to the burden that any trial paperwork has on care home staff and how to ensure experiences relevant to an oral health intervention are captured. Indeed, time poverty and the competing needs of care home staff, as well as staff characteristics and turnover, were key challenges for the intervention delivery identified through the parallel process evaluation [38], and these factors are also in line with other relevant studies [25, 29]. On the other hand, intervention records were completed for most residents in the intervention arm, with high completion rates for the oral care plans. This supports the feasibility of the intervention. Moreover, TOPIC was a pragmatic feasibility study, and the delivery of the oral health intervention was based on the commitment of care homes, allowing them to embed it in their routine without necessarily specifying that this should be delivered by one specific member of the staff. This was facilitated by access to the training package throughout the study and including it in new staff induction, making its implementation potentially less affected by staff turnover. A detailed account about intervention fidelity is presented separately [38]. The number of protocol deviations was also limited, suggesting that a definitive study is possible, notwithstanding the caveats mentioned above. There were many adverse events (affecting 1/6 of those recruited), though none of them was related to the study, and this could potentially increase further in a subsequent definitive trial, particularly if residents with severe cognitive impairment are included, as seems appropriate from the discussion above.

Exploratory analysis showed quite modest and unlikely to be clinically meaningful differences in most oral health measures, including coronal caries, root caries, levels of visible plaque, and the OHAT, as expected with the small sample size. There were somewhat larger differences in relation to the proportion of sites that bled on probing and oral health‐related quality of life, with residents in the intervention arm having lower levels of gingival bleeding and reporting lower OIDP scores (i.e., better quality of life) than those in the control arm at the 12‐month follow‐up. The proportion of sites with bleeding on probing is a measure of oral inflammation, while OIDP reflects the perceptions of residents about the impact oral conditions have on their daily life. Such variables may be more sensitive to this intervention and therefore better suited as outcomes for a preventive oral health intervention based primarily on oral hygiene, though whether such differences would be clinically meaningful needs to be established. Improvement in residents' perceptions about their quality of life is a key consideration in the context of healthy ageing, and even more so for people living in care homes where dignity, maintaining their identity and participating in daily life activities determine their well‐being [51, 52, 53]. Oral health related quality of life was also seen as an important outcome from the care home managers' perspective [39]. Given the nature of the intervention, a definitive study would need to be based on a cluster design to minimise contamination. Thus, the estimates of intra‐cluster correlation coefficients are also important, and these varied considerably across the different measures. This is a feasibility study and therefore not powered to assess clinical effectiveness but provides preliminary information to guide the consideration of an appropriate primary outcome measure with which to power a definitive trial. To have generalisable findings, such a future trial would need to be conducted in different settings in the UK and cover the wide range of care homes features (e.g., in terms of size, organisation, resident characteristics).

This feasibility study has contributed to the limited empirical evidence‐base for interventions to improve oral health among dependent older adults in care homes. In a recent review, only twelve of the thirty included experimental studies were identified as having a low risk of bias [30]. Of these, only five studies were based on a cluster design and apart from one study [31], the remaining definitive studies [32, 33, 34, 49] recruited a smaller number of care homes than this study. Using a theoretically informed co‐design approach to refine the oral health intervention based on the NICE NG48 guideline resulted in intervention materials that were grounded in the experience of older people in care homes and those that provide their care. This facilitated ‘a good fit’ between intervention and context and is particularly relevant for public health interventions [54, 55]. At the same time, delivering the study in the volatile care home environment proved challenging. Care home staff and management commitment were essential while frequent management changes negatively affected recruitment and retention. This was amplified due to the COVID‐19 pandemic as there were several outbreaks hindering the recruitment of care homes and residents despite allowing for a 10‐month recruitment period. Moreover, the recovery from these outbreaks was gradual and varied by setting and care home, meaning that study arrangements were further delayed with consequences on retention rates. This provided important learnings in terms of engagement and adopting a flexible and pragmatic approach for planning ahead a larger trial. Maintaining links with the care homes, engaging throughout the process with the funding body (NIHR) and receiving advice and support from relevant oversight groups (i.e., the Study Steering Committee and the Data Monitoring and Ethics Committee) and from the NIHR Clinical Research Networks and the NHS Community Dental Services have helped keep the study on track through these prolonged and unprecedented challenges.

In conclusion, this study of an oral health intervention in care homes showed that recruitment and retention of both care homes and residents was feasible, while data completion and intervention recording was successful. These suggest that a definitive trial could be undertaken to assess the effectiveness of the co‐designed intervention, but more inclusive recruitment, high retention, minimising missing data and outcome selection are important issues to consider.

## Author Contributions

G.T., G.M. and P.R.B. conceived the study and together with Z.H., R.G.W., R.R.W., A.S., C.J.S., F.K., C.O.N., A.H., J.L., P.C., and N.L. were responsible for the study design. S.S., M.H., S.D., S.W., and A.V. were responsible for the running of the study and acquisition of the data. N.G. was responsible for data analysis, and all authors were responsible for interpretation. G.T. prepared the first draft of the manuscript, and G.T., G.M., P.R.B., and S.S. revised it following considerable input from the whole team. The final version of the manuscript was approved by all authors.

## Ethics Statement

The TOPIC study was approved by the London: City & East Research Ethics Committee (ref: 19/LO/1107).

## Consent

All participants provided written informed consent.

## Conflicts of Interest

The authors declare no conflicts of interest.

## Supporting information


**Table S1.** Characteristics of residents at baseline.

## Data Availability

The data that support the findings of this study are available from the corresponding author upon reasonable request.
