# Supplementary material for: Improving the Oral Health of Older People in Care Homes: Results From a Randomised Feasibility Study
Source: Community Dent Oral Epidemiol. 2025 Jun 10;53(4):413–23. doi: 10.1111/cdoe.13043 (PMC12238747; doi:10.1111/cdoe.13043)
Supplement: Supplementary file 1 — Table S1. Characteristics of residents at baseline. [file CDOE-53-413-s001.docx]

**Supplementary Table 1. Characteristics of residents at baseline.**

| Characteristic | Descriptive | Control | Intervention | Total |
| --- | --- | --- | --- | --- |
| Age (years) | N (Missing) | 63 (1) | 52 (3) | 115 (4) |
|  | Mean (SD) | 85.1 (8.0) | 81.9 (9.3) | 83.6 (8.7) |
|  | Median [IQR] | 85 [81, 91] | 82 [75, 88.5] | 85 [78, 90] |
|  | Min, Max | 62, 102 | 65, 99 | 62, 102 |
| Gender | Male | 21 (32.8%) | 24 (43.6%) | 45 (37.8%) |
|  | Female | 42 (65.6%) | 28 (50.9%) | 70 (58.8%) |
|  | Missing | 1 (1.6%) | 3 (5.5%) | 4 (3.4%) |
| Ethnicity | English/Welsh/Scottish/Northern Irish/British/Irish | 56 (87.5%) | 46 (83.6%) | 102 (85.7%) |
|  | Any other white background | 2 (3.1%) | 2 (3.6%) | 4 (3.4%) |
|  | White and Black Caribbean | 2 (3.1%) | 0 (0%) | 2 (1.7%) |
|  | Indian | 2 (3.1%) | 1 (1.8%) | 3 (2.5%) |
|  | African | 1 (1.6%) | 1 (1.8%) | 2 (1.7%) |
|  | Caribbean | 0 (0%) | 1 (1.8%) | 1 (0.8%) |
|  | Any other ethnic group | 0 (0%) | 1 (1.8%) | 1 (0.9%) |
|  | Missing | 1 (1.6%) | 3 (5.5%) | 4 (3.4%) |
| Marital Status | Single (never married) | 13 (20.3%) | 10 (18.2%) | 23 (19.3%) |
|  | Married / civil partnership | 8 (12.5%) | 7 (12.7%) | 15 (12.6%) |
|  | Separated | 1 (1.6%) | 3 (5.5%) | 4 (3.4%) |
|  | Divorced | 3 (4.7%) | 7 (12.7%) | 10 (8.4%) |
|  | Widowed | 37 (57.8%) | 25 (45.5%) | 62 (52.1%) |
|  | Missing | 2 (3.1%) | 3 (5.5%) | 5 (4.2%) |
| Previous Employment | Professional and managerial | 22 (34.4%) | 15 (27.3%) | 37 (31.1%) |
|  | Clerical and Sales | 18 (28.1%) | 15 (27.3%) | 33 (27.7%) |
|  | Skilled blue-collar | 12 (18.8%) | 7 (12.7%) | 19 (16.0%) |
|  | Semi-skilled and unskilled | 6 (9.4%) | 8 (14.5%) | 14 (11.8%) |
|  | Other | 2 (3.1%) | 7 (12.7%) | 9 (7.6%) |
|  | Prefer not to say | 3 (4.7%) | 0 (0%) | 3 (2.5%) |
|  | Missing | 1 (1.6%) | 3 (5.5%) | 4 (3.4%) |
| 6-CIT score | N (Missing) | 63 (1) | 51 (4) | 114 (5) |
|  | Mean (SD) | 4.5 (2.9) | 3.4 (3.1) | 4.0 (3.0) |
|  | Median [IQR] | 4 [2, 8] | 2 [0, 6] | 4 [2, 6] |
|  | Min, Max | 0, 9 | 0, 9 | 0, 9 |
